# A d-peptide-based oral nanotherapeutic modulates the PD-1/PD-L1 interaction for tumor immunotherapy

**DOI:** 10.3389/fimmu.2023.1228581

**Published:** 2023-07-17

**Authors:** Dan Liu, Jingmei Wang, Weiming You, Fang Ma, Qi Sun, Junjun She, Wangxiao He, Guang Yang

**Affiliations:** ^1^ Department of General Surgery, First Affiliated Hospital of Xi’an Jiaotong University, Xian, China; ^2^ Department of Medical Oncology, The First Affiliated Hospital of Xi’an Jiaotong University, Xi’an, China; ^3^ Department of Talent Highland, The First Affiliated Hospital of Xi’an Jiaotong University, Xi’an, China; ^4^ Institute for Stem Cell & Regenerative Medicine, The Second Affiliated Hospital of Xi’an Jiaotong University, Xi’an, China; ^5^ National & Local Joint Engineering Research Center of Biodiagnosis and Biotherapy, The Second Affiliated Hospital of Xi’an Jiaotong University, Xi’an, China; ^6^ Department of Oncology, Kunshan Hospital of Chinese Medicine, Affiliated Hospital of Yangzhou University, Yangzhou, China

**Keywords:** immunotherapy, peptide, supramolecular nanospheres, milk exosome, anti-tumor

## Abstract

**Background:**

PD-1/PD-L1 immune checkpoint inhibitors are currently the most commonly utilized agents in clinical practice, which elicit an immunostimulatory response to combat malignancies. However, all these inhibitors are currently administered *via* injection using antibody-based therapies, while there is a growing need for oral alternatives.

**Methods:**

This study has developed and synthesized exosome-wrapped gold–peptide nanocomplexes with low immunogenicity, which can target PD-L1 and activate antitumor immunity *in vivo* through oral absorption. The ^Super^PDL1^exo^ was characterized by transmission electron microscopy (TEM), dynamic light scattering (DLS), Fourier transform infrared (FTIR), X-ray photoelectron spectroscopy (XPS), and gel silver staining. The transmembrane ability of ^Super^PDL1^exo^ was evaluated by flow cytometry and immunofluorescence. Cell viability was determined using the Cell Counting Kit-8 (CCK-8) assay. ELISA experiments were conducted to detect serum and tissue inflammatory factors, as well as serum biochemical indicators. Tissue sections were stained with H&E for the evaluation of the safety of ^Super^PDL1^exo^. An MC38 colon cancer model was established in immunocompetent C56BL/6 mice to evaluate the effects of ^Super^PDL1^exo^ on tumor growth *in vivo*. Immunohistochemistry (IHC) staining was performed to detect cytotoxicity factors such as perforin and granzymes.

**Results:**

First, ^Super^PDL1 was successfully synthesized, and milk exosome membranes were encapsulated through ultrasound, repeated freeze–thaw cycles, and extrusion, resulting in the synthesis of ^Super^PDL1^exo^. Multiple characterization results confirmed the successful synthesis of ^Super^PDL1^exo^ nanoparticles. Furthermore, our data demonstrated that ^Super^PDL1^exo^ exhibited excellent colloidal stability and superior cell transmembrane ability. *In vitro* and *in vivo* experiments revealed that ^Super^PDL1^exo^ did not cause damage to multiple systemic organs, demonstrating its good biocompatibility. Finally, in the MC38 colon cancer mouse model, it was discovered that ^Super^PDL1^exo^ could inhibit the progression of colon cancer, and this tumor-suppressive effect was mediated through the activation of tumor-specific cytotoxic T lymphocyte (CTL)-related immune responses.

**Conclusion:**

This study has successfully designed and synthesized an oral nanotherapeutic, ^Super^PDL1^exo^, which demonstrates small particle size, excellent colloidal stability, transmembrane ability in tumor cells, and biocompatibility. *In vivo* experiments have shown that it effectively activates T-cell immunity and exerts antitumor effects.

## 1 Introduction

Immunotherapy has been playing an important role in the treatment of cancer with the development of drugs that target immune checkpoints (1–4). In the tumor microenvironment, immune checkpoint blockade therapy that targets PD-1 and PD-L1 may reawaken T lymphocytes specialized for tumors, may break the tumor immune tolerance mechanism that has already been established in the body, and has shown significant clinical benefits in cancer, including non-small cell lung cancer (NSCLC), melanoma, colorectal cancer, renal cell carcinoma (RCC), breast cancer, bladder cancer, and Hodgkin’s lymphoma (5–8).

However, the total response rate to anti-PD-1/PD-L1 immunotherapy has been modestly approximately 30% (9). In addition, some patients who initially responded to αPD-1 and αPD-L1 therapy have subsequently shown tumor recurrence or drug resistance (10). The occurrence of immune-related adverse events (irAEs), in particular, is a contributing factor in immunotherapy failures. Additionally, the therapeutic effect of antibody medicines may be negated by compensatory upregulation of PD-L1 within tumor cells, active redistribution of PD-L1 to the cell membrane, and the consumption of antibodies by PD-L1 in tumor exosomes at distant regions of the tumor (11–13). Given the difficulties that PD-1 and PD-L1 inhibitors face, it is vital to look at novel strategies to enhance antitumor immunotherapy.

Peptides, which are composed of several to tens of natural or non-natural amino acids, can be obtained through natural product extraction, genetic recombination, chemical synthesis, etc. They have shown significant therapeutic effects in cancer, bacterial infections, diabetes, osteoporosis, multiple sclerosis, HIV infection, chronic pain, immune diseases, etc. (14, 15). Moreover, peptide drugs with low immunogenicity, easy synthesis and modification, and good tissue penetration are being considered as alternatives to antibodies, which can reduce the occurrence of adverse reactions in immunotherapy effectively. In this study, we synthesize P-peptide using Fmoc-protected d-amino acids as a raw material to target and disrupt the function of PD-L1 in tumor cells, with a standard solid-phase synthesis method. Although peptide drugs have shown good efficacy, tolerability, and safety, the indications for approved antitumor peptide drugs are relatively limited. The main reasons for this limitation are their short half-life in the body, susceptibility to proteolytic degradation, poor physicochemical stability, and low membrane permeability, which hinder their stable therapeutic effects and targeting of intracellular targets (14). In addition, peptide drugs are currently administered *via* intravenous injection, intramuscular injection, subcutaneous injection, etc., which have disadvantages such as poor patient compliance, risk of accidental injury and infection, improper use, and improper disposal of biologically hazardous needles. Compared to these routes of administration, oral administration is more convenient and feasible, is non-invasive, allows for greater dose flexibility and self-administration, and has higher patient compliance (16). However, the oral bioavailability of peptides is low, and it is also a challenge to find a reasonable and effective oral strategy for the development of peptide drugs. Therefore, effective improvements and optimizations in peptide drug delivery and pharmaceutical chemistry are necessary.

With the development of nanotechnology, peptides can overcome inherent barriers. Research has shown that peptide-derived nanotechnologies, including peptide-based polymeric nanoparticles, peptide-coated nanoparticles, and peptide-based self-assembled nanostructures, possess promising biological benefits such as resistance to protein degradation and cell membrane permeability (17–21). Due to their inherent inertness, minimal cytotoxicity, and affordability, gold nanoparticle-conjugated peptides have become more explored and employed in clinical studies for the delivery of drugs and biomolecules. In order to avoid the treatment failure resulting from compensatory upregulation of PD-L1 inside tumor cells, gold nanoparticle-conjugated peptides were further designed and synthesized and then self-assembled into ^super^PD-L1 supramolecular nanospheres under the mild reducing conditions created by HEPES. Our previous studies have shown that these gold-derived peptide gold nanoparticles have higher loading efficiency (22).

Furthermore, milk exosomes were chosen as the membrane encapsulation for gold nanoparticle-conjugated peptide supramolecular nanospheres, which could be efficiently absorbed orally. Milk exosomes are evolutionarily conserved nanovesicles naturally contained in milk. Recent studies have shown that milk exosomes can withstand the harsh acid–base environment in the gastrointestinal tract and maintain the structural and functional stability of the nucleic acids and proteins they contain while passing through the gut (23). Due to their low immunogenicity, excellent stability, and capacity to pass through the gastrointestinal barrier, milk exosomes are a potential oral delivery vehicle.

Here, this work provided a nano-gold peptide supramolecular nano-candidate (^Super^PD-L1^exo^) for tumor immunotherapy and, more importantly, offered a feasible approach for optimizing peptide performance for oral administration. The drug was proven to possess excellent tumor cell penetration ability and colloidal stability and demonstrate good biosafety both *in vitro* and *in vivo*. In addition, ^Super^PD-L1^exo^ might stimulate tumor immunity and deliver excellent antitumor effects when taken orally.

## 2 Results

### 2.1 Synthesis of P-peptide and extraction of milk exosomes

**Figure 1:**
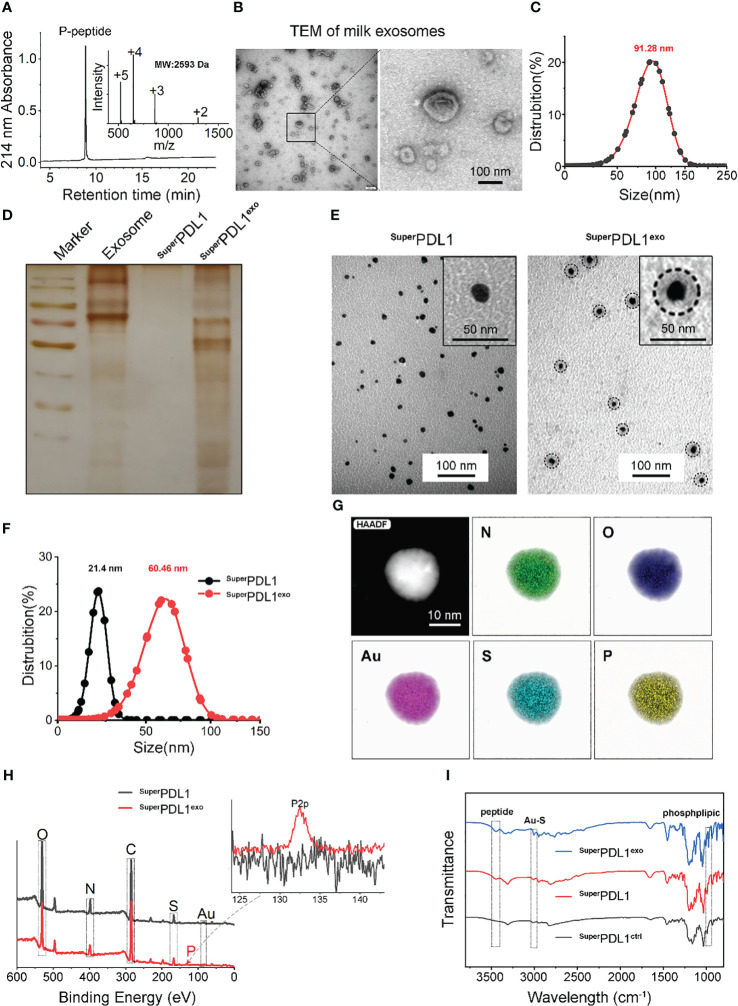
Synthesis and characterization of ^Super^PDL1^exo^. **(A)** ^Super^PDL1^exo^ analyzed by high-performance liquid chromatography (HPLC) and electrospray ionization mass spectrometry (ESI-MS). **(B, C)** Negative staining transmission electron micrograph (TEM; B) image and hydrodynamic diameter **(C)** of milk exosomes. **(D)** Protein detection in membrane of milk exosomes, ^Super^PDL1, and ^Super^PDL1^exo^ by sodium dodecyl sulfate–polyacrylamide gel electrophoresis (SDS-PAGE) with silver stain. **(E)** TEM images of ^Super^PDL1 and ^Super^PDL1^exo^. **(F)** Hydrodynamic diameter distributions of ^Super^PDL1 and ^Super^PDL1^exo^ measured in phosphate-buffered saline (PBS) buffer at pH 7.4. **(G)** The elemental analysis image of N, O, Au, S, and P overlay with one representative particle of ^Super^PDL1^exo^ taken by high-resolution TEM (HRTEM). **(H)** Energy-dispersive X-ray spectrum of ^Super^PDL1 and ^Super^PDL1^exo^. **(I)** Comparison of the Fourier transform infrared (FTIR) spectra of ^Super^PDL1^exo^, ^Super^PDL1, and ^Super^PDL1^ctrl^.

PD-L1 is upregulated in many cancers and shields tumor cells from T cell-mediated immune surveillance and killing. Inhibiting the function of PD-L1 can reactivate antitumor immunity. We first synthesized P-peptide, which can inhibit the function of PD-L1. P-peptide was synthesized using solid-phase peptide synthesis with d-amino acids protected by fluorene methoxyl (Fmoc) upon HBTU/HOBT-catalyzed condensation reaction (24–26). Mass spectrometry indicated the relative molecular weight of the P-peptide to be 2,593 Da, and then high-performance liquid chromatography (HPLC) confirmed its purity (**Figure 1A**), indicating its successful synthesis. In addition, the fluorescein isothiocyanate (FITC) was conjugated to the N-terminus of P-peptide. Flow cytometry analysis showed that, compared to the control group, the P-peptide-treated group exhibited minimal uptake, indicating a weaker internalization capability of P-peptide into tumor cells (**Figure S1**).

Transmission electron microscopy (TEM) and dynamic light scattering (DLS) were applied to characterize the exosomes after they were extracted from fresh milk by ultracentrifugation. As described in **Figure 1B**, typical “saucer-like” cup-shaped exosomes with double-layered membrane structures, ranging from 30 to 150 nm, were observed in TEM micrographs to be negatively stained with uranyl acetate. DLS further confirmed the size distribution of the milk exosomes (**Figure 1C**), indicating successful extraction of milk exosomes.

### 2.2 Synthesis and characterization of ^Super^PDL1

**Figure 2:**
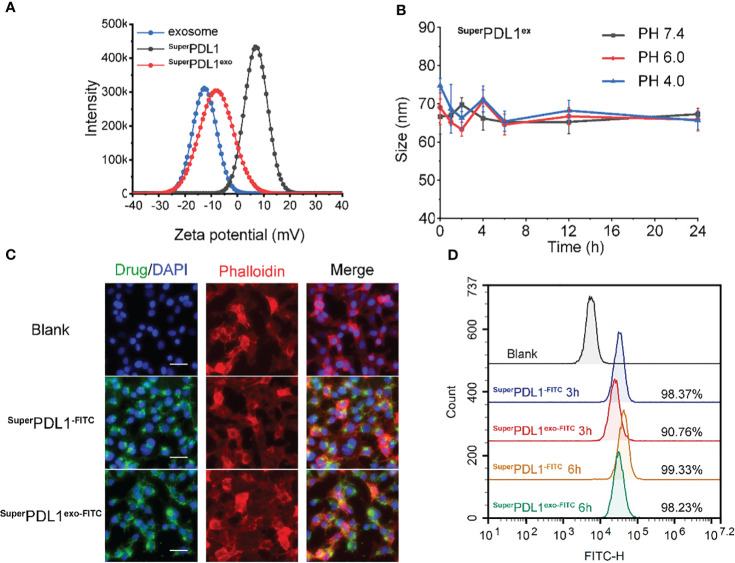
*In vitro* stability and cell internalization profiles of ^Super^PDL1^exo^. **(A)** Zeta potential distribution of milk exosomes, ^Super^PDL1, and ^Super^PDL1^exo^ measured by dynamic light scattering. **(B)** Colloidal stability of ^Super^PDL1^exo^ suspending in phosphate-buffered saline (PBS) containing 20% fetal bovine serum (FBS) at pH 4.0, 6.0, and 7.4 measured by dynamic light scattering (DLS). **(C)** Confocal laser scanning microscopy (CLSM) images of MC38 cells after 6 h of incubation with fluorescein isothiocyanate (FITC)-loading ^Super^PDL1 and ^Super^PDL1^exo^. The cytoskeletal phalloidin is marked in red. Nuclei were stained with DAPI (blue). All images were taken under the same excitation light and detector gain (scale bar, 100 μm). **(D)** Cellular uptakes of FITC-loading ^Super^PDL1 and ^Super^PDL1^exo^ into MC38 cells measured by flow cytometry after 3- and 6-h incubations.

As we previously reported, thiols-N-terminally-modified peptides could form a comonomer precursor *via* infinite Auric-sulfhydryl coordination and then self-assembled into spherical nanostructures under the advantage of aurophilicity (20, 22, 27, 28). First, P-peptide was dissolved under the reducing conditions of NH2-PEG-SH and absolute ethanol to form P-peptide-SH, and then with the addition of Au^3+^, the thiols in P-peptide-SH reduced Au^3+^ to form a comonomer precursor of [Au(I)-S-P peptide]n, which further self-assembled into ^Super^PDL1 nanospheres through aurophilicity. ^Super^PDL1 was observed by TEM to have a uniform size distribution, a shape that was close to spherical, and a diameter of approximately 20 nm and exhibited good monodispersity (**Figure 1E**). The average hydrodynamic diameter of ^Super^PDL1 measured by the DLS experiment is 21.4 nm, with a narrow peak distribution, showing reasonable size uniformity (**Figure 1F**), which was consistent with the TEM results. In addition, the surface potential (ζ-potential) of ^Super^PDL1 was 6.84 mV (**Figure 2A**). Overall, these results demonstrated that ^Super^PDL1 has been successfully constructed as a spherical supramolecular Au(I)-SH-P-peptide complex.

### 2.3 Synthesis and characterization of ^Super^PDL1^exo^

**Figure 3:**
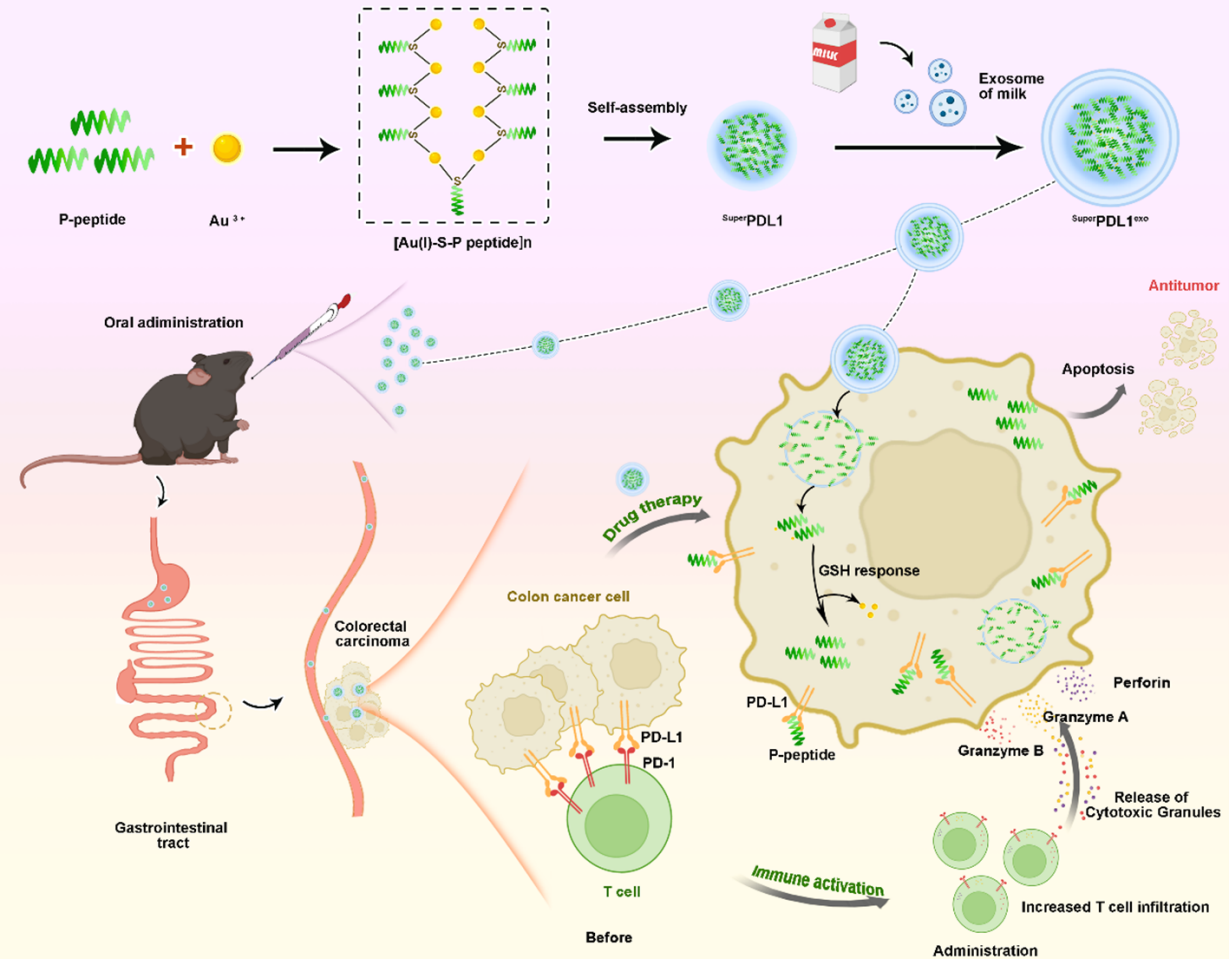
A schematic of the synthetic procedure and colorectal cancer therapy of ^Super^PDL1^exo^. First, the designed P-peptide that modulates the PD-1/PD-L1 interaction, along with Au3^+^ and thiol peptides, was used to synthesize the [Au(I)-S-P peptide]_n_ precursor. Subsequently, under mild reducing conditions created by HEPES, the self-assembly of [Au(I)-S-P peptide]_n_ occurred, forming the ^Super^PD-L1 supramolecular nanospheres. Finally, the ^Super^PDL1^exo^, capable of oral administration, was synthesized by coating with milk exosome membranes, which effectively inhibited tumor growth by blocking the PD-1/PD-L1 interaction, activating CD8^+^ T-cell immunity in a mouse model of colorectal cancer.

To confer the ^Super^PDL1 nanospheres with more biological properties improving the physical stability and stability in the gastrointestinal tract, the milk exosome membrane was wrapped around the surface of ^Super^PDL1 through ultrasonication, repeated freeze–thaw cycles, and extrusion to form the final product, ^Super^PDL1^exo^ (**Figure 3**). To confirm if the exosome membrane was successfully wrapped around the surface of ^Super^PDL1, the proteins in ^Super^PDL1^exo^ were examined using sodium dodecyl sulfate–polyacrylamide gel electrophoresis (SDS-PAGE) silver staining. As illustrated in **Figure 1D**, the proteins of ^Super^PDL1^exo^ (line 4) were similar to the expression of milk exosome membrane protein (line 2), whereas no proteins were detected in ^Super^PDL1 (line 3). This result indicated the successful wrapping of the milk exosome membrane. As expected, ^Super^PDL1^exo^ was observed through TEM to be uniformly distributed in size, with particles close to spherical with a diameter of approximately 42 nm, and exhibiting good monodispersity (**Figure 1E**). Further, elemental analysis of ^Super^PDL1^exo^ by high-resolution TEM (HRTEM) illustrated the uniform distribution of nitrogen (N), oxygen (O), gold (Au), sulfur (S), and phosphorus (P) (**Figure 1G**), which showed that Au and P-peptide were evenly distributed in the ^Super^PDL1^exo^ nanoparticles, and the structure of the original ^Super^PDL1 was not destroyed during the synthesis of ^Super^PDL1^exo^. The presence of the P element also indicated the successful wrapping of the milk exosome membrane. The average hydrodynamic diameter of ^Super^PDL1^exo^ increased to 60.46 nm as measured by DLS, further supporting the successful encapsulation of the exosome membrane (**Figure 1F**). The size of the micelle assessed by DLS was larger compared to that obtained by TEM, which could be attributed to different testing environments. During DLS testing, ^Super^PDL1^exo^ particles were in a hydrated state and expansion, while during TEM testing, they were in a completely dry and dehydrated state, resulting in a significant reduction in particle size (29). Additionally, energy-dispersive X-ray spectroscopy (EDS) testing revealed that ^Super^PDL1^exo^’s uniform distribution of composition elements was consistent with that of chloroauric acid, peptides, and exosomes (**Figure 1H**). These results were further verified by the Fourier transform infrared (FTIR) spectra in **Figure 1I**. All these demonstrated that ^Super^PDL1 had been successfully assembled into ^Super^PDL1^exo^.

DLS analysis showed that the membrane of the exosomes carried a negative charge, which is consistent with previous reports (15, 30). The wrapping of the membrane caused a charge to flip from a positive of ^Super^PDL1 to a negative (−8.50 mV) of ^Super^PDL1^exo^, which is similar to the charge of the exosome membrane (−12.64 mV), further confirming the successful synthesis of ^Super^PDL1^exo^ (**Figure 2A**). The change in surface charge of ^Super^PDL1^exo^ may facilitate its rapid absorption through the mucous layer in the intestine. Studies have shown that surface charge affects the speed of particles passing through the gastrointestinal mucous layer, thereby affecting their absorption efficiency. Particles with a positive or neutral charge move slowly through the mucous layer due to their electrostatic interaction with mucin, while particles with a negative charge can quickly move and be absorbed (31). These findings provide sufficient evidence to demonstrate the successful construction of the supramolecular complex called ^Super^PDL1^exo^, and it is worth noting that the wrapping of the milk exosome membrane did not change the spherical morphology and composition of ^Super^PDL1.

### 2.4 Structural stabilities of ^Super^PDL1^exo^

To test whether the synthesized final product, ^Super^PDL1^exo^ nanoparticles, remained stable in blood circulation, they were suspended and diluted in phosphate-buffered saline (PBS) solutions containing 20% fetal bovine serum (FBS) at three different values of pH (4.0, 6.0, and 7.4). The fluid dynamic diameter of ^Super^PDL1^exo^ was continuously monitored using DLS during 24 h of incubation. The results showed that ^Super^PDL1^exo^ remained monodisperse, and the hydrodynamic diameter did not change significantly (**Figure 2B**). This indicated satisfactory colloidal stability, demonstrating that ^Super^PDL1^exo^ had the required structural stability under simulated physiological conditions and could maintain its integrity in blood circulation. After entering the bloodstream, the surface of the nanoparticles may be adsorbed by non-specific proteins to form a protein corona, a phenomenon known as protein adsorption. Protein corona formation may reduce the targeting and delivery functionality of nanoparticles, and its formation is related to the physicochemical properties of the nanoparticle surface (32). This study observed that there was no significant increase of ^Super^PDL1^exo^ in hydrodynamic diameter after being incubated in PBS containing 20% FBS at different pH values, indicating its structural stability.

### 2.5 Cellular uptakes and biodistribution

FITC was attached to the N-terminus of P-peptide to prepare FITC-labeled ^Super^PDL1 and ^Super^PDL1^exo^, and the uptake of ^Super^PDL1 and ^Super^PDL1^exo^ by colon cancer cells was qualitatively observed using confocal laser scanning microscopy (CLSM), and quantitatively analyzed using flow cytometry. Blue, green, and red respectively represent DAPI, FITC, and Cy3 fluorescence signals (Cy3-labeled phalloidin cytoskeleton). **Figure 2C** shows the results of confocal laser scanning microscopy imaging, which demonstrated that ^Super^PDL1 and ^Super^PDL1^exo^ had strong penetration ability of colon cancer cells after co-incubation for 6 h. Furthermore, compared to ^Super^PDL1, the penetration ability of the exosome membrane-wrapped ^Super^PDL1^exo^ did not significantly weaken.

The findings of the flow cytometry analysis are shown in **Figure 2D**, demonstrating that ^Super^PDL1 and ^Super^PDL1^exo^ were able to enter more than 90% of the tumor cells at 3 h, with percentages of 98.37% and 90.76%, respectively, showing higher tumor cell penetration ability. Although the internalization ability of ^Super^PDL1^exo^ was slightly weaker than that of ^Super^PDL1, it gradually accumulated with prolonged incubation time. At 6 h, the FITC signals of both ^Super^PDL1 and ^Super^PDL1^exo^ were enhanced and approached saturation. These results indicated that ^Super^PDL1^exo^ designed and synthesized in this study has a strong ability to internalize tumor cells, laying the foundation for its antitumor effect.

To investigate the biodistribution characteristics of ^Super^PDL1^exo^, we quantified ^197^Au by inductively coupled plasma mass spectrometry (ICP-MS) to detect the distribution of ^Super^PDL1^exo^ and ^Super^PDL1 in C57/B6 mice with subcutaneous MC38 tumors. After 4 h of oral administration, the accumulation of ^Super^PDL1^exo^ in the liver, spleen, kidney, lung, and tumor was significantly higher than that of ^Super^PDL1, indicating superior intestinal absorption (**Figure S2**). This finding was further supported by a significant decrease in the concentration of ^Super^PDL1^exo^ in the intestine at 12 and 24 h after oral administration (**Figure S2**). Furthermore, ^Super^PDL1^exo^ exhibited superior tumor accumulation when compared to ^Super^PDL1 at all time points (**Figure S2**).

### 2.6 Cytotoxicity study of ^Super^PDL1^exo^

**Figure 4:**
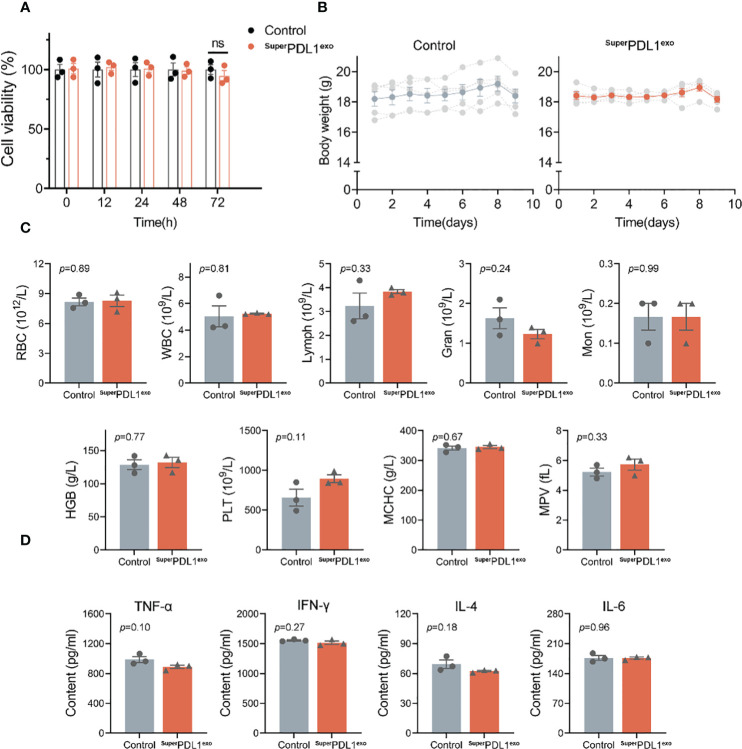
The safety evaluation of ^Super^PDL1^exo^. **(A)** Cell viability of MC38 cells in response to ^Super^PDL1^exo^ treatment at different time points (12, 24, 48, and 72 h) measured by Cell Counting Kit-8 (CCK-8) assay. Healthy C57BL/6 mice were administered with saline (as control) or ^Super^PDL1^exo^ (10 mg/kg, an average of five doses) and then weighed, and blood was collected on day 9 to assess the safety. **(B)** Variations of body weight of C57BL/6 mice with different treatments over time. **(C)** Hematological parameters of the mice after 9 days of the indicated treatment. Red blood cells (RBC), white blood cells (WBC), lymphocytes (Lymph), granulocyte (Gran), monocyte (Mon), hemoglobin (HGB), MCHC, platelets (PLT), and mean platelet volume (MPV). **(D)** Concentrations of cytokines (TNF-α, IFN-γ, IL-4, and IL-6) in serum when compared between saline and ^Super^PDL1^exo^-treated C57BL/6 mice detected by ELISA. Values are represented as mean ± SEM.

Excellent biosafety is a prerequisite for the transformation of nanomedicines into the clinic. Therefore, we validated whether ^Super^PDL1^exo^ had cytotoxicity *in vitro*. As shown in **Figure 4A**, ^Super^PDL1^exo^ had no impact on the viability of MC38 cells after incubation for 12, 24, 48, and 72 h as measured by Cell Counting Kit-8 (CCK-8) assay. Consistent results were also obtained on normal colonic epithelial cells (NCM460), indicating that ^Super^PDL1^exo^ was not cytotoxic (**Figure S3**).

### 2.7 Biosafety evaluation of ^Super^PDL1^exo^ *in vivo*

Immune checkpoint inhibitors (ICIs) may result in a variety of inflammatory adverse effects, or irAEs, when they disinhibited T-cell activity (4). To evaluate the *in vivo* safety of ^Super^PDL1^exo^, the body weight and blood parameters of mice were first monitored after administration. C57BL/6 mice in the ^Super^PDL1^exo^ group were administered orally with ^Super^PDL1^exo^ every other day for five doses at a dosage of 2 mg/kg per mouse. An equivalent dose of saline solution was used as a control (n = 5/group). Every day, mouse body weights were recorded. In the end, the major organs (heart, lung, liver, kidney, and spleen) were removed and extracted from the mice for histological examination. Blood from mice was collected for ELISA and hematological testing. **Figure 4B** demonstrates that there was no discernible loss in mouse body weight following continuous oral administration of ^Super^PDL1^exo^ compared to the healthy control. The small drop in total body weight on the last day was caused by fasting before sample collection. Additionally, there were no significant variations between the two groups in the blood cell counts of white blood cells, red blood cells, monocytes, neutrophils, lymphocytes, hemoglobin, platelets, and related parameters, which were within the normal reference range, indicating no adverse effects of ^Super^PDL1^exo^ such as infection, hemolysis, or thrombocytopenia (**Figure 4C**). Furthermore, serum inflammatory cytokine detection was used to reflect whether ^Super^PDL1^exo^ would induce systemic allergic inflammation. As shown in **Figure 4D**, there was no increase in serum inflammatory cytokine amounts of TNF-α, IL-4, IFN-γ, and IL-6 in the ^Super^PDL1^exo^-treated group, indicating that ^Super^PDL1^exo^ had good biocompatibility and did not cause serious immune inflammatory reactions.

**Figure 5:**
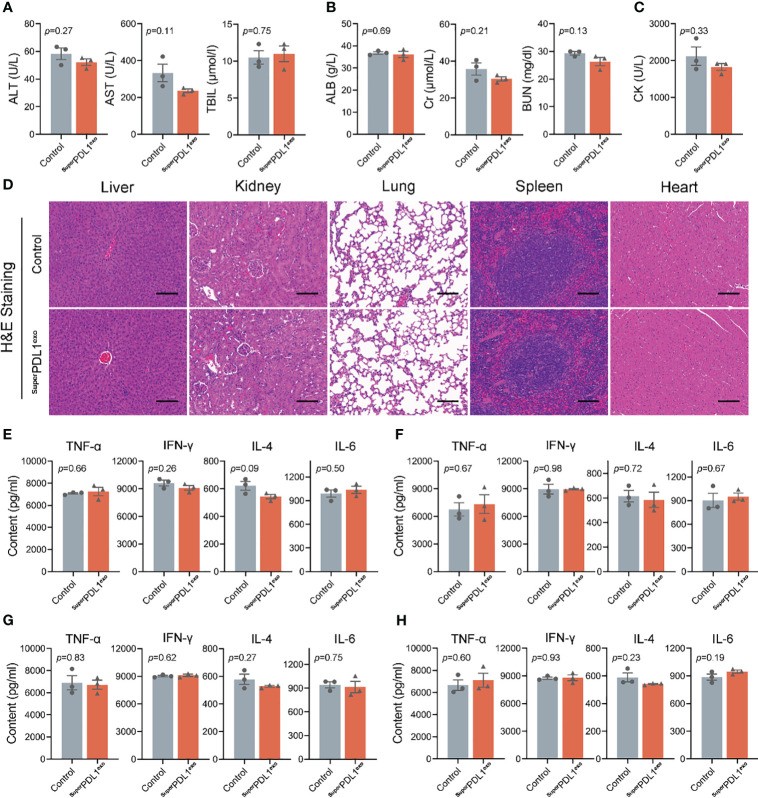
Toxicity detection of ^Super^PDL1^exo^ on the major organs *in vivo*. Immune-competent C57BL/6 mice were administered intragastrically with phosphate-buffered saline (PBS) or ^Super^PDL1^exo^, and the major organs were collected at the end of the experiment to assess the toxicity and immunogenicity. **(A)** Activities of liver enzymes related to liver function in healthy mice after indicated treatments, including alanine transaminase (ALT), aspartate aminotransferase (AST), and total bilirubin (TBIL). **(B)** The evaluation of renal function indicators in mice with the indicated treatments. ALB, albumin; CR, serum creatinine; BUN, blood urea nitrogen. **(C)** The detection of serum creatine kinase (CK) was used to reflect myocardial injury. **(D)** Representative images of H&E-stained pathological sections of mouse liver, kidney, lung, spleen, and heart after the dosing (scale bar, 100 μm). **(E-H)** ELISA to quantify the inflammatory factors infiltrated in liver, lung, kidney, and spleen tissue: TNF-α, IFN-γ, IL-4, and IL-6. Each point represents the mean ± SEM (n = 3).

After entering the bloodstream, drugs pass through various organs and then are metabolized and excreted from the body. Subsequently, we evaluated whether ^Super^PDL1^exo^ would cause potential damage to organs by monitoring changes in a series of indicators in serum, tissue slices, and tissue homogenates. Alanine transaminase (ALT), aspartate aminotransferase (AST), total bilirubin (TBIL), Cr, and blood urea nitrogen (BUN) levels are commonly used to assess liver and kidney damage. An increase in ALT, AST, Cr, and BUN levels indicates impaired function of the liver and kidney (33, 34). According to this experiment’s findings, there were no appreciable changes in ALT, AST, TBIL, albumin (ALB), Cr, and BUN levels between the ^Super^PDL1^exo^-treated group and the control group (**Figures 5A, B**). Therefore, it can be inferred that as an oral nanoparticle, ^Super^PDL1^exo^ did not cause damage to the liver and kidney. Furthermore, as shown in **Figure 5C**, serum creatine kinase (CK) testing indicated that ^Super^PDL1^exo^ did not cause myocardial damage. According to H&E staining analysis, slices of the liver, kidney, lung, spleen, and heart tissue did not exhibit any histopathological damage (**Figure 5D**), further indicating that ^Super^PDL1^exo^ had no obvious toxic and side effects on major organs. In addition, no significant differences were observed in inflammatory cytokine amounts in tissue homogenates of liver (**Figure 5E**), lung (**Figure 5F**), kidney (**Figure 5G**), and spleen (**Figure 5H**), suggesting that ^Super^PDL1^exo^ did not cause inflammatory damage to organs.

The intestine is the main site for digestion and absorption of nutrients and is the first line of defense against harmful substances in the human body. Therefore, we investigated the toxicity of continuous administration on intestinal tissues. During the administration, mice did not exhibit vomiting, anorexia, diarrhea, or weight loss. After continuous administration, gastric and intestinal tissues were stained with H&E, and the results showed no pathological damage to the stomach, small intestine, or colon, indicating good gastrointestinal safety (**Figure S4**). Overall, the changes in body weight, blood parameters, organ function, and section staining demonstrated that oral administration of ^Super^PDL1^exo^ had good biosafety, providing the possibility for further clinical translation research.

### 2.8 ^Super^PDL1^exo^ vastly improves the anticancer efficacy *in vivo*

**Figure 6:**
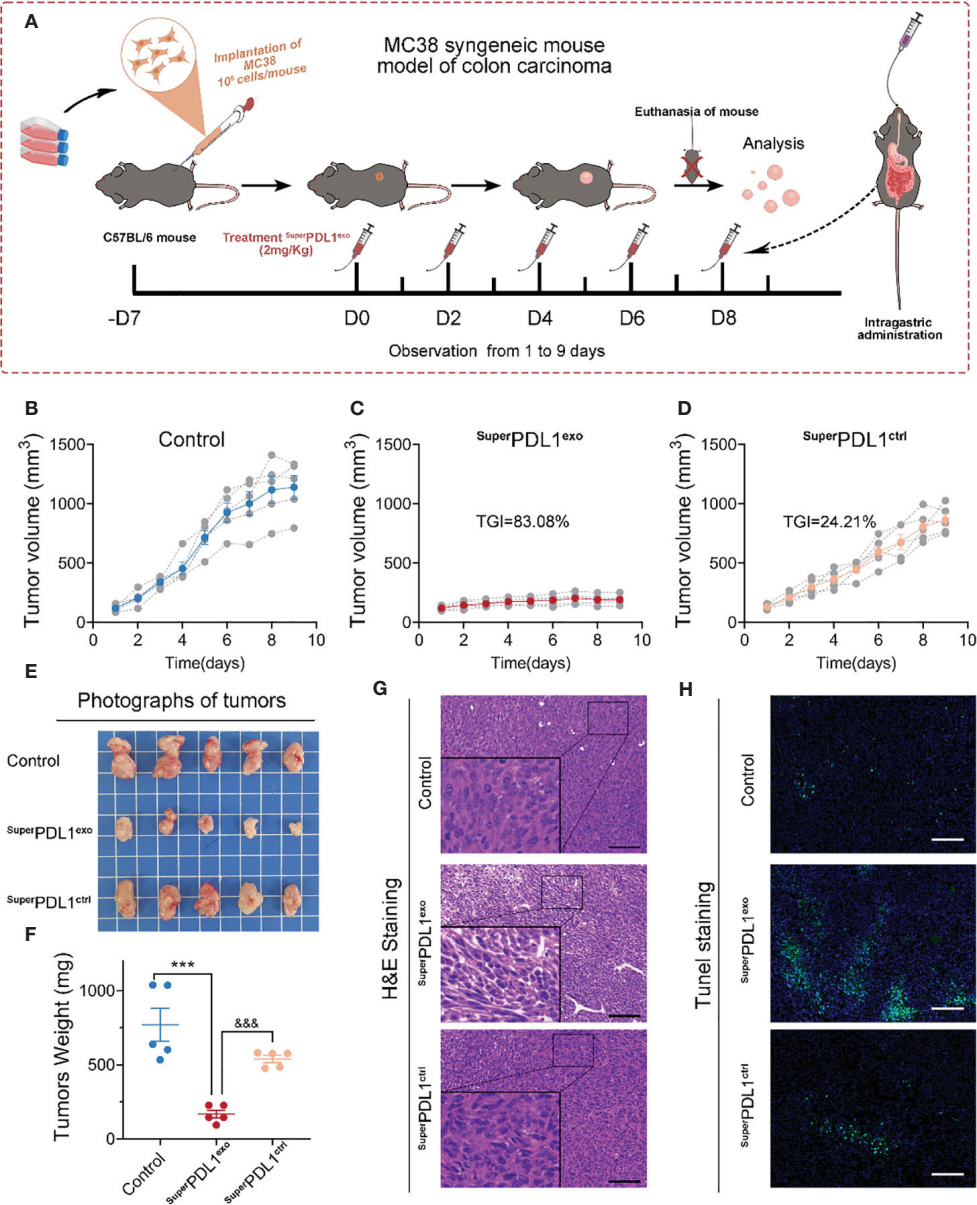
^Super^PDL1^exo^ potently suppressed the tumor progression in C57BL/6 mice implanted with MC38 syngeneic colon carcinoma. **(A)** Diagrammatic sketch of mouse model with indicated treatments. C57BL/6 mice bearing MC38 (10^6^ cells/mouse) colorectal tumors in the right flank were established and randomly divided into three groups when the volume of tumors reached 50–100 mm^3^ (n = 5/group). Mice then received an oral administration of saline (control) or 2 mg/kg of ^Super^PDL1^exo^ or ^Super^PDL1^ctrl^ five times every other day. ^Super^PDL1^ctrl^ was a control synthesized in the same system as ^Super^PDL1^exo^ but without the P-peptide component. **(B-D)** Tumor growth curves of control group **(B)**, ^Super^PDL1^exo^ group **(C)**, or ^Super^PDL1^ctrl^ group **(D)**. **(E, F)** Representative photographs **(E)** and mass **(F)** of tumor dissection harvested in each group at the end of treatment. *p*-Values were calculated by *t*-test (***/&&& *p* < 0.001. ***, ^Super^PDL1^exo^ *vs.* control; &&&, ^Super^PDL1^exo^ *vs.* ^Super^PDL1^ctrl^). **(G, H)** Representative images of H&E **(G)** and TUNEL **(H)** staining in MC38 tumor sections from C57BL/6 mice after indicated treatments (scale bar, 100 μm).

Encouraged by the excellent colloidal stability, tumor cell membrane permeability, and superior *in vivo* biosafety mentioned above, the MC38 mouse colon adenocarcinoma model was used to evaluate the antitumor efficacy of ^Super^PDL1^exo^ in immunocompetent C57BL/6 mice (MC38 cells have been reported to exhibit high PD-L1 expression levels (11)). As shown in **Figure 6A**, to evaluate the effect of ^Super^PDL1^exo^ on colon cancer, C57BL/6 mice bearing MC38 (10^6^ cells/mouse) colorectal tumors in the right flank were established and randomly divided into three groups when the volume of tumors reached 50–100 mm^3^ (n = 5/group). After that, mice were intragastrically administered with normal saline (Control) or 2 mg/kg of ^Super^PDL1^exo^ or ^Super^PDL1^ctrl^ five times every other day. The mice were euthanized when the tumor volume arrived at approximately 1,500 mm^3^, and then the tumors were isolated for further study. ^Super^PDL1^ctrl^ was a control synthesized in the same system as ^Super^PDL1^exo^ but without the P-peptide component. As observed in **Figures 6B–D**, ^Super^PDL1^exo^ had a great inhibitory effect on tumor growth (83.08%) as compared with the control group, while ^Super^PDL1^ctrl^ had only 24.21% tumor growth inhibition ability. The tumor photos (**Figure 6E**) and tumor weights (**Figure 6F**) further confirmed the above conclusion. **Figure 6G** shows the H&E-stained tumor tissue slices from each treatment group. Terminal deoxynucleotidyl transferase dUTP nick end labeling (TUNEL) staining showed that tumor cell apoptosis has been dramatically enhanced in the ^Super^PDL1^exo^ group compared with the saline or ^Super^PDL1^ctrl^ groups (**Figures 6H**, **S5**). These findings indicated that ^Super^PDL1^exo^ has superior antitumor effects *in vivo*.

### 2.9 ^Super^PDL1^exo^ suppressed tumor progression by activating antitumor immunity

**Figure 7:**
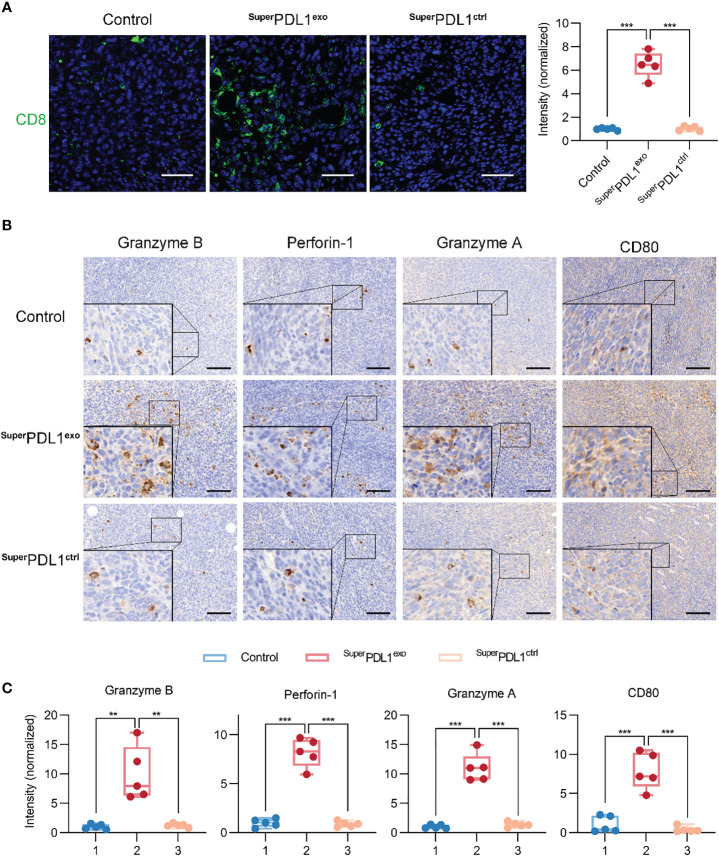
^Super^PDL1^exo^ effectively activates tumor immunity. **(A)** Immunofluorescence staining images and quantification of CD8 of tumor after administration (scale bar, 50 μm). **(B, C)** Representative images **(B)** and the scores **(C)** of immunohistochemistry (IHC) analysis of granzyme B, perforin-1, granzyme A, and CD80 in tumor section from mice with different treatments (scale bar, 100 μm; *** *p* < 0.001, ** *p* < 0.01).

PD-L1 can inhibit T-cell proliferation and cytolytic activity by interacting with its receptor PD-1 on immune cells, helping cancer cells escape immune surveillance. To investigate the targeting and disruption effects of ^Super^PDL1^exo^ on PD-L1, we performed immunohistochemical analysis to determine the abundance of PD-L1 as shown in **Figure S6**. Compared to the control and ^Super^PDL1^ctrl^ groups, the ^Super^PDL1^exo^ group exhibited a statistically significant downregulation of PD-L1 expression. To explore whether ^Super^PDL1^exo^ exerts antitumor effects by activating the antitumor immunity as expected, the infiltration of CD8+ cytotoxic T lymphocytes (CTLs) and a series of immune-related cytotoxic granules in tumors were then examined. First, the tissue immunofluorescence staining of CD8 was used to assess the extent of tumor CTL infiltration and showed that ^Super^PDL1^exo^ markedly boosted the infiltration of CD8+ T cells (**Figure 7A**). Through their T-cell receptor (TCR), CTLs recognize antigen-MHC complexes on tumor cells and induce tumor cell apoptosis by secreting perforin and granzyme, with perforin opening a channel in the target cell membrane and granzymes entering the cytoplasm to trigger an enzyme chain reaction leading to cell death (35). In this study, the perforin, granzyme B, and granzyme A secreted by CTL cells in tumor tissue were evaluated by immunohistochemical staining. As illustrated in **Figures 7B, C**, ^Super^PDL1^exo^ dramatically increased the abundance of perforin, granzyme B, and granzyme A. Additionally, studies have shown that PD-L1 can bind to the costimulatory molecule CD80 produced on T cells, sending an inhibitory signal (36). When CD80 expression in tumor tissues was examined by immunohistochemistry (IHC) staining, it was observed that the expression of CD80 increased after the ^Super^PDL1^exo^ treatment. All these data showed that ^Super^PDL1^exo^ played an antitumor role by activating CTL-mediated tumor immunity.

## 3 Discussion

Immune-related adverse events and compensatory upregulation of PD-L1 within tumor cells are the main reason for immunotherapy failures. Here, we designed and synthesized an oral ^Super^PDL1^exo^ supramolecular nanomedicine to activate tumor T-cell immunity and inhibition of tumor growth. First, based on peptide-conjugated gold self-assembling nanostructures, the ^Super^PDL1 was designed and synthesized, which could target PD-L1 on both the cell membrane and inside the cell, which resulted from its attractive tumor cell penetration ability. Moreover, ^Super^PDL1 with a smaller size can non-specifically accumulate in tumor tissues based on passive targeting, which can effectively reduce the occurrence of immune-related adverse events.

Then, we successfully extracted high-quality milk exosomes. Consistent with previous reports (37–39), the milk exosomes observed under TEM and DLS had a diameter of 30–150 nm and a lipid bilayer structure that appeared circular or cup-shaped. ^Super^PDL1 was coated with milk exosome membrane to further synthesize ^Super^PDL1^exo^ by repeated freezing and thawing, ultrasonication, and extrusion. The success of the exosome membrane wrapped was confirmed by TEM imaging, DLS particle size increase, high-resolution imaging element analysis, X-ray photoelectron spectroscopy (XPS), and FTIR of ^Super^PDL1^exo^. SDS-PAGE gel silver staining results of the milk exosomes, ^Super^PDL1, and ^Super^PDL1^exo^ samples showed that ^Super^PDL1^exo^ had similar protein components to milk exosomes, further supporting this result. Milk exosomes have been proven to be a potential natural drug delivery system for delivering drugs, therapeutic proteins, nucleic acids, peptides, and targeting ligands due to their scalability and economic feasibility. Furthermore, Zhong et al. have found that milk exosomes have the ability to stimulate the proliferation of intestinal epithelial cells and regulate immunity (23). Studies also have shown that milk exosomes loaded with curcumin can withstand breakdown by human digestive enzymes and have increased intestinal permeability *in vitro* (40), and camouflage chiral peptides with milk exosomes effectively facilitate their absorption from the gut into circulation and thus play a role (27).

We have demonstrated that in a C57BL/6 mouse model of colorectal cancer, ^Super^PDL1^exo^ is a supramolecular nanosphere that could be absorbed orally and can effectively inhibit tumor growth, compared with the control group and the empty vector Control–^Super^PDL1^ctrl^. Oral administration is a powerful strategy for therapeutic delivery in cancer therapy, which is a non-invasive and safe method that allows for greater dose flexibility and self-administration, helping to improve patient compliance. The particle diameter of ^Super^PDL1^exo^ with the negative charge was much smaller than 200 nm, which had shown a good foundation for subsequent oral absorption and reaching target organs in our study.

In this study, under physiological conditions simulated with fetal bovine serum *in vitro*, the particles of ^Super^PDL1^exo^ remained in a monodisperse state and had almost no change in size during continuous monitoring for 24 h, which was not without the influence of exosome membrane wrapping. The prolongation of the drug’s time in the bloodstream can facilitate the tissue-targeted delivery of the drug. There are membrane-bound proteins and transmembrane proteins (CD63, CD9, CD81, etc.) on the surface of the exosome membrane, which could effectively prolong the blood circulation time (23).

^Super^PDL1^exo^ was synthesized by Au-loaded polypeptides while being enveloped by milk exosome membranes, where gold had a great advantage in terms of safety due to its chemical inertness (41–43); P-peptides were formed by the condensation of multiple amino acids, and milk exosomes were naturally occurring nanovesicles with low immunogenicity. We examined the toxicity of ^Super^PDL1^exo^ *in vitro* and *in vivo* and evaluated its toxicity to major organs through a series of blood indexes, serum biochemical indexes, and tissue H&E staining, demonstrating that ^Super^PDL1^exo^ had good biosafety. Furthermore, there was no significant change in serum inflammatory factors and major organ inflammatory factors, indicating that it did not cause immunotoxicity. The evidence for the involvement of the microbiome in cancer treatment is growing, and studies have revealed that the gut microbiome specifically affects how different types of cancer respond to immune checkpoint blockade (44–47). It is worth further investigating whether the orally administered nanodrug ^Super^PDL1^exo^ that we constructed affects the stability of the gut microbiota.

​During the administration of PD-1/PD-L1 immune checkpoint inhibitors, tumors may compensatively upregulate PD-L1 expression, which subsequently leads to acquired resistance to PD-1/PD-L1 blocking, limiting the sustainability of anti-PD-1/PD-L1 efficacy. To counteract this resistance mechanism, the nano-system ^Super^PDL1^exo^ constructed by us was designed to target the total PD-L1 expressed in tumor cells, rather than the PD-L1 expressed on the membrane surface. Our data showed that compared to the control group, ^Super^PDL1^exo^ could enhance infiltration of CD8^+^ T cells in tumors; increase the expression of cytotoxic granules such as perforin, granzyme B, and granzyme A; and exert antitumor effects in the MC38 colon cancer model. However, during the development of resistance to anti-PD-1/PD-L1 therapy, in addition to compensatory PD-L1 upregulation, there were other resistance mechanisms (48, 49). It may be worth considering the simultaneous reversal of multiple resistance mechanisms within the same delivery system to enhance antitumor immunity, such as improving T-cell initiation by increasing antigen presentation (50). Additionally, the contribution of intrinsic oncogenic signals to PD-1/PD-L1 blockade resistance should not be overlooked (51, 52).

## 4 Conclusion

In summary, we designed an oral immunotherapy supramolecular nanoparticle, ^Super^PDL1^exo^, which is based on the self-assembly of gold nano-peptides wrapped by exosome membranes. ^Super^PDL1^exo^ could effectively interact with cancer cells through gastrointestinal absorption after oral administration, with features such as circulatory stability, efficient cell membrane penetration, and high biocompatibility. Safety evaluations *in vitro* and *in vivo* confirmed that ^Super^PDL1^exo^ has no cytotoxicity and systemic side effects. Importantly, ^Super^PDL1^exo^ demonstrated significant activation of tumor T-cell immunity and inhibition of tumor progression in an immune-competent colorectal cancer mouse model. We believe that this oral nanomedicine can be effectively applied to a variety of solid tumor types, offering a meaningful approach for triggering powerful immune responses against cancer.

## Data availability statement

The raw data supporting the conclusions of this article will be made available by the authors, without undue reservation.

## Ethics statement

The animal study was reviewed and approved by The medical ethics committee of Xi’an Jiaotong University.

## Author contributions

DL and JW was responsible for the study design. DL, JW, WY, FM and QS conducted experiments. WH and GY were responsible for writing the article. DL, JW, WY, FM and QS was responsible for data acquisition and processing analysis. JS was responsible for data checking and interpretation. All authors contributed to the article and approved the submitted version.
