# Supplementary material for: A d-peptide-based oral nanotherapeutic modulates the PD-1/PD-L1 interaction for tumor immunotherapy
Source: Front Immunol. 2023 Jul 17;14:1228581. doi: 10.3389/fimmu.2023.1228581 (PMC10388715; doi:10.3389/fimmu.2023.1228581)
Supplement: Supplementary file 1 [file DataSheet_1.docx]

**Supplementary materials**

**A D-peptide-based oral** **nanotherapeutic modulates the PD-1/PD-L1 interaction for tumor immunotherapy**

**Dan Liu^1,2,^**^†^**, Jingmei Wang^3,^**^†^**, Weiming You^4^,** **Fan Ma^4^, Qi, Sun^1^, Junjun She^1*^, Wangxiao He^2,3,*^, Guang Yang^5*^**

^1^Department of General Surgery, First Affiliated Hospital of Xi’an Jiaotong University, Xian 710061, China

^2^Department of Medical Oncology and Department of Talent Highland, The First Affiliated Hospital of Xi’an Jiaotong University, Xi’an 710061, PR. China.

^3^Institute for Stem Cell & Regenerative Medicine, The Second Affiliated Hospital of Xi’an Jiaotong University, Xi’an 710004, China

^4^National & Local Joint Engineering Research Center of Biodiagnosis and Biotherapy, The Second Affiliated Hospital of Xi'an Jiaotong University, Xi'an, 710004, PR. China.

^5^Department of Oncology, Kunshan Hospital of Chinese Medicine, Affiliated Hospital of Yangzhou University, Yangzhou, 225009, China

^†^ These authors contributed equally.

^*^ Corresponding authors:

Email: [junjunshe1975@sina.com](mailto:junjunshe1975@sina.com) (J. She)

Email: [hewangxiao5366@xjtu.edu.cn](mailto:hewangxiao5366@xjtu.edu.cn) (W. He)

E-mail: yangguang9002@163.com (G. Yang)

**Supplementary Figures**

**
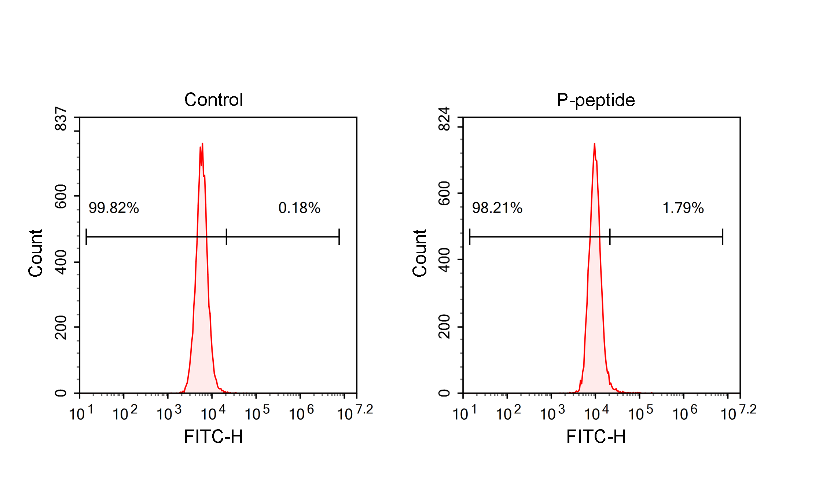
**

**Figure S1.** Cellular uptakes of FITC-loading P-peptide into MC38 cells measured by flow cytometry after 6 h incubations.


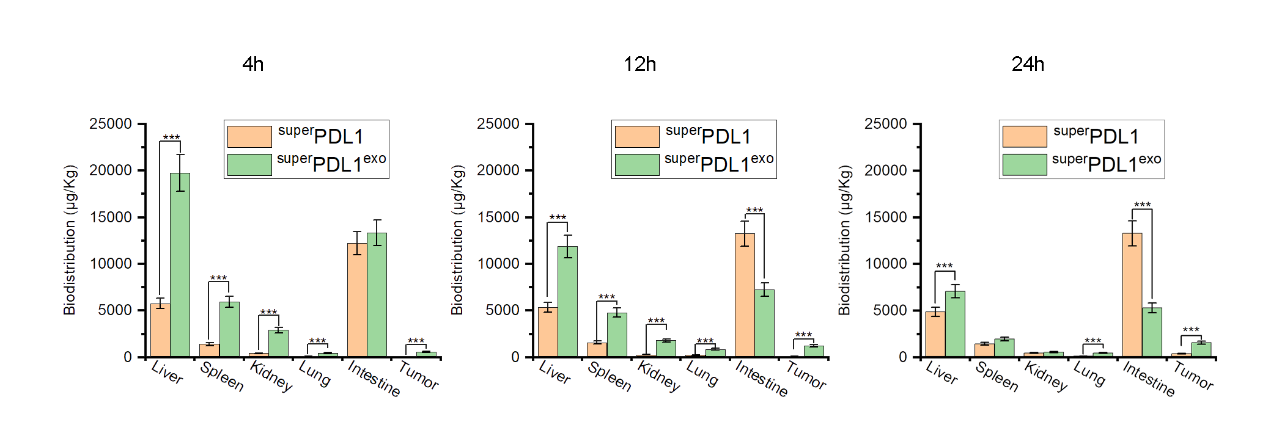


**Figure S2.** Biodistribution of ^Super^PDL1^exo^ and ^Super^PDL1 through the quantification of 197Au by ICP-MS at 4h, 12h and 24h after oral medication.

**
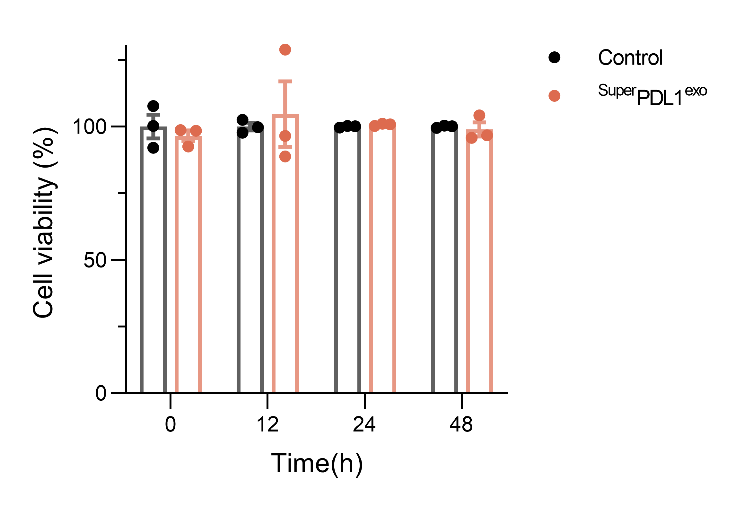
**

**Figure S3.** Cell viability of NCM460 cells in response to ^Super^PDL1^exo^ treatment measured by CCK8 assay.


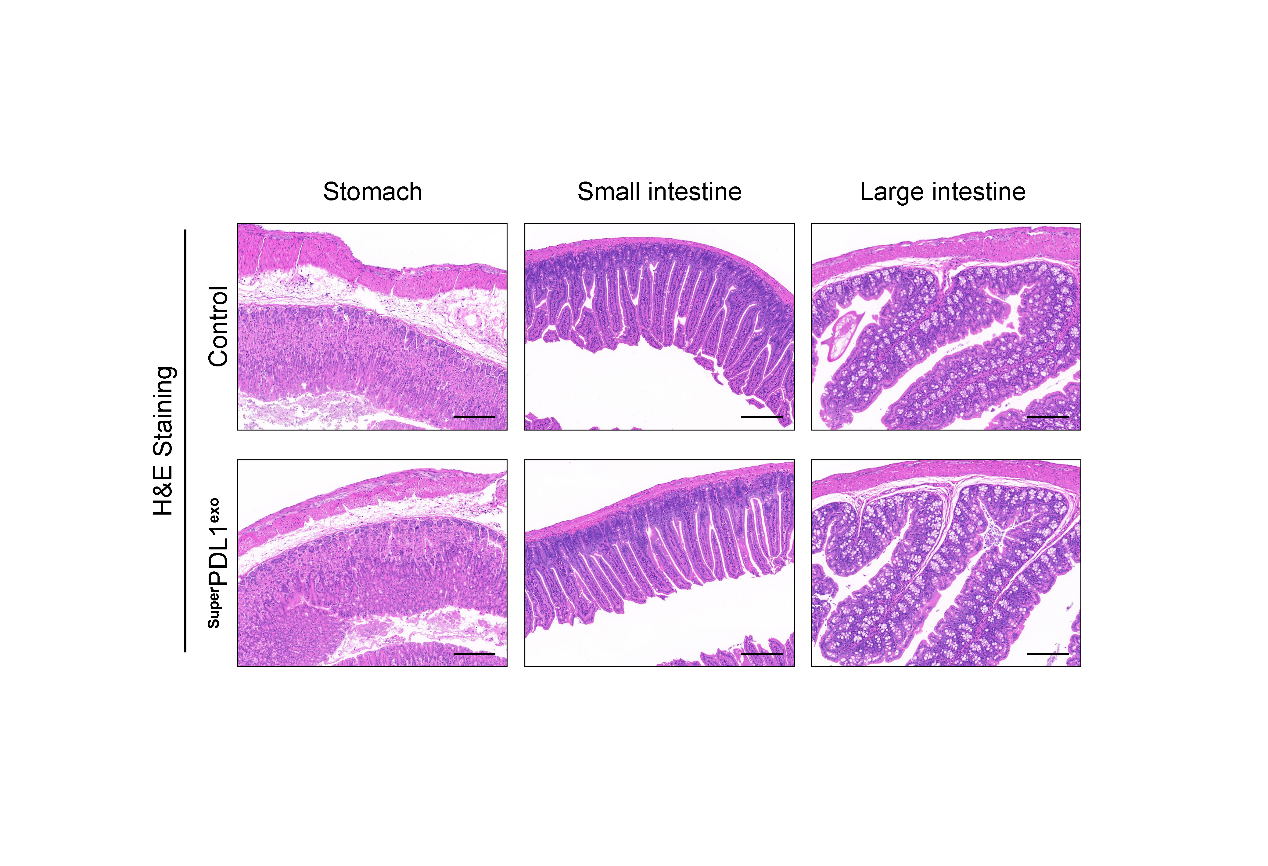


**Figure S4.** Representative images of H&E-stained pathological sections of mouse stomach, small intestine, and small intestine after administration. (Scale bar: 200 μm).


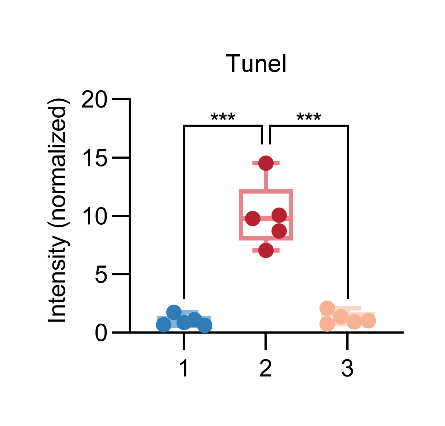


**Figure S5.** Cell viability of MC38 cells in response to ^Super^PDL1^exo^ treatment at different time points (12 h, 24 h, 48 h, and 72 h) measured by CCK8 assay. (*** *p* < 0.001)

**
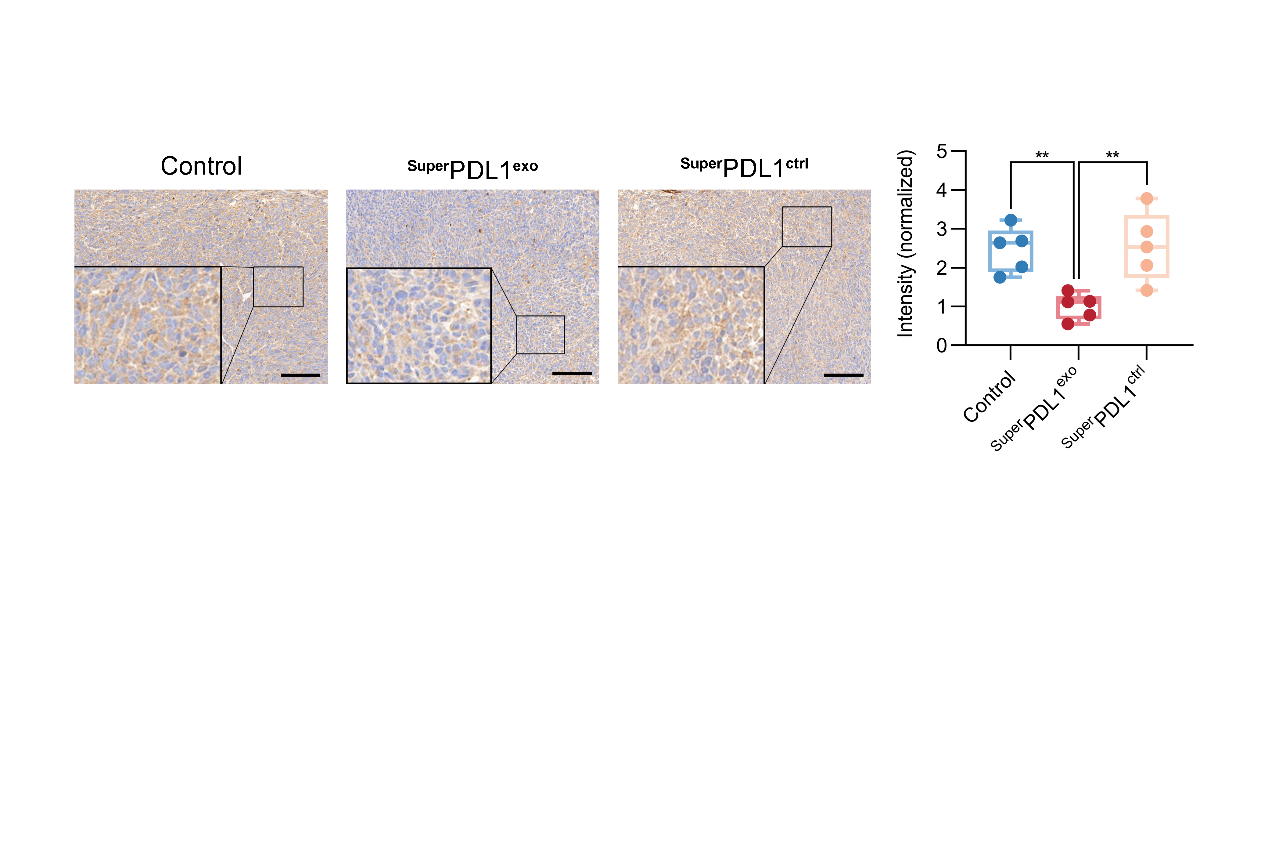
**

**Figure S6.** Representative IHC image of PD-L1 and IHC score (scale bar:100 μm, ** *p* < 0.01).

**Materials and methods**

**1 Materials**

All sources of synthetic peptide were purchased from CS Bio Ltd. All other compounds used in this study, unless specified otherwise, were bought from Sigma-Aldrich. Without additional purification, all products utilized in their original form.

**2 Preparation of ^Super^PDL1^exo^**

Firstly, P-peptide was synthesized from D-amino acid by solid phase polypeptide synthesis method based on Fmoc (N-9-fluorene methoxycarbonyl). Exosomes were extracted from fresh milk by ultrafast centrifugation. After that, 2 mg P-peptide was completely dissolved in a solution containing 500 μL NH_2_-PEG-SH (4 mg/mL) and 500 μL anhydrous ethanol. Then 1.25 mL deionized water (ddH_2_O), 2.25 mL HEPES (pH 7.0) and 500 μL aqueous solution of tetrachloroauric acid (HAuCl_4_·XH_2_O) were added, mixed and reacted for a period of time. A mixture containing 500 μL HAuCl_4_·XH_2_O, 2.25 ml HEPES (pH 7.0) and 2.25 ml ddH_2_O was added, stirred at 50 °C and ultrasonicated for 10 minutes. The product was subsequently washed twice with dd-water before the excess reactants were removed using dialysis tubing (cutoff, 10 KDa). Here, ^Super^PDL1 of the 10 ml system was synthesized.

Subsequently, the exosomes prepared by ultrasonic crushing were added to the synthetic ^Super^PDL1. After repeated freezing and sonication, ^Super^PDL1 mixed with milk exosome membranes was extruded through 200-nm polycarbonate porous membranes for multiple cycles to form ^Super^PDL1^exo^.

**3 Characterization of ^Super^PDL1^exo^**

P-peptide was identified by ESI-Mass electro ionization mass spectrometry. Sample solution was suspended on a 200-mesh carbon film copper net and dried for TEM morphologies. Using high-resolution transmission electron microscopy (HRTEM), the nanoparticles' morphology lattice structure, and elemental composition were examined. At 25 °C, the dynamic light scattering (DLS) was applied to evaluate the zeta potential and particle size distribution of ^Super^PDL1 and ^Super^PDL1^exo^. Fourier transform infrared (FT-IR) spectroscopy (Brooke VERTEX 70) was used to assess the surface chemical structure of nanocrystals.

**4 Characterization of milk exosomes**

DLS worked to assess the hydrodynamic nanoparticle size and surface zeta potential of milk exosomes. The exosomes were stained with 0.2 weight percent uranyl acetate for imaging and observed under a transmission electron microscope.

**5 Colloidal stability of ^Super^PDL1^exo^**

For stability characterization, ^Super^PDL1^exo^ were suspended and diluted into PBS solution containing 20% fetal bovine serum (FBS) at three pH of 4.0, 6.0 and 7.4. During the incubation period of 24 h, the hydrodynamic diameter of ^Super^PDL1^exo^ was continuously measured by DLS.

**6 Cells culture**

RPMI 1640 medium (Gibico, USA) with 10% fetal bovine serum (FBS, Gibico, USA) was used to cultivate MC38 cells (murine colon carcinoma cell line), which were obtained from the cell bank of the National Collection of Authenticated Cell Cultures (Shanghai, China). The cells were cultured at 37 °C, 5% CO_2_.

**7 MC38 cellular uptake experiments**

MC38 cells were incubated with FITC-loading ^Super^PDL1 and ^Super^PDL1^exo^ at a predetermined concentration and time. Briefly, MC38 cells were plated in 6-well dishes until 80% confluence was reached. Then FITC-loading ^Super^PDL1 and ^Super^PDL1^exo^ (10 μg. mL^-1^) were incubated with MC38 cells for 3 hours or 6 hours. After digested and centrifuged, the cells were collected into 1.5 ml ep tubes. After three times washed with PBS (pH 7.4), they were subjected to Flow cytometry analysis.

Meanwhile, cells that had been co-incubated with ^Super^PDL1 and ^Super^PDL1^exo^ (10 μg. mL^-1^) for 6 h were observed under an inverted fluorescence microscope. Firstly, crawler tablets of MC38 cell were prepared in 6-well plates and incubated with FITC-loading ^Super^PDL1 and ^Super^PDL1^exo^ after 24 h of cell adhesion. To get rid of free FITC-loaded ^Super^PDL1 and ^Super^PDL1^exo^, the media was aspirated followed which the cells were washed three times in PBS. The MC38 cells on the crawler tablets were then fixed using 4% paraformaldehyde and stained with phalloidin that had been Cy3-labeled. DAPI was used to stain the nuclei. Finally, the fluorescence intensities of FITC and Cy3 were captured using CLSM.

**8 *In vitro* biosafety experiments**

Through the CCK8 assay, the *in vitro* cytotoxicities of ^Super^PDL1^exo^ against MC38 cells were assessed. In 96-well plates, MC38 cells were planted with a density of 3×10^3^ cells / well and allowed to attach for 24 hours. The incubator was then changed to RPMI 1640 complete medium containing ^Super^PDL1^exo^ (10 μg. mL^-1^) and incubated for a different amount of time. The cells were subsequently incubated for another 2 hours at 37 °C in medium containing 10 μL of CCK-8. The relative cell vitality (%) was calculated by the absorbance of ^Super^PDL1^exo^-treated comparing to that of untreated control wells that set at 450 nm.

**9 Animals**

Female C57BL/6 mice weighing between 18 and 20 g and aged 6 to 8 weeks were procured from the experimental animal center of Xi’an Jiaotong University (Xi’an, China). Mice were housed with standard chow and typical light/dark cycles under standard specific pathogen-free (SPF) conditions. Before the experiment, the mice were given seven days to get adapted to the environment. All animal procedures were conducted in according to ethical regulations for animal research and were approved by the medical ethics committee of Xi’an Jiaotong University.

**10 Biosafety of ^Super^PDL1^exo^ *in vivo***

10 healthy C57 mice were randomized into two groups (n = 5) and given 2 mg.kg^-1^ of ^Super^PDL1^exo^ or saline (Control) every other day orally. The body weight of the mice in each group was measured daily. After receiving the medication continuously for five cycles, the mice were sacrificed and gathered the blood for examining the hematological parameters and serum biochemical index. Meanwhile, the heart, liver, lung, kidney and spleen tissues were also acquired. Some of them were homogenized to detect inflammatory factors by ELISA (TNF-α, IL-4, IFN-γ, and IL-6), others were fixed and then stained with Hematoxylin and Eosin (H&E) to assess the security and immunogenicity of the preparations.

**11 *In vivo* antitumor experiments**

C57BL/6 mice bearing MC38 (10^6^ cells/mouse) colorectal tumors into the right flank were constructed and randomly assigned into three groups when the volume of tumors reached 50–100 mm^3^ (n = 5 /group). Saline (Control), 2 mg.kg^-1^ ^Super^PDL1^exo^ or ^Super^PDL1^ctrl^ were then administered orally to mice five times every other day. The volume of tumors was recorded every day after administration and calculated by caliper expressing as the formula 1/2×length×width^2^. When the volume of tumor reached 1500 mm^3^, each group mice were sacrificed, and then the tumor was dissected and its weight was recorded. After fixed specimens in 10% formalin phosphate-buffered solution, the tissues were paraffin embedded and 3 μm paraffin sections were used for staining. Furthermore, H&E staining and Granzyme B, Perforin-1, Granzyme A and CD80 immunohistochemistry assay were performed on the tumors to examine the anti-tumor effect of ^Super^PDL1^exo^.

**12 Statistical Analysis**

All quantitative data were presented as mean ± SEM. Unless otherwise stated, GraphPad Prism 9.0 software was used to assess statistical differences between groups using a two-tailed Student's t-test. * *p* < 0.05 was judged statistically significant.
